# The role of poverty-related social determinants in maternal and perinatal health inequities: a cross-sectional study using the eLIXIR born in South London, UK maternity-child data linkage

**DOI:** 10.1186/s12939-026-02793-3

**Published:** 2026-03-14

**Authors:** Sam Burton, Tisha Dasgupta, Zenab Barry, Abigail Easter, Jane Sandall, Hannah Rayment-Jones

**Affiliations:** 1 School of Psychology, Liverpool John Moores University, Liverpool, UK; 2 Department of Women and Children’s Health, King’s College London. London, London, UK

## Abstract

**Background:**

Evidence on how poverty and social determinants influence adverse maternal and perinatal outcomes in the UK is limited. While ethnicity and area-level deprivation are well described, fewer studies examine the cumulative impact of poverty-related factors such as low income, employment insecurity, housing, and access to social support.

**Methods:**

We analysed 67,308 pregnancies from the eLIXIR cohort using linked NHS records. Social determinants were defined using the WHO framework as structural (ethnicity, migration status, area deprivation) and intermediary (housing, employment, financial hardship, social support, barriers to care). The primary outcome was a composite adverse perinatal outcome. Binary logistic regression models with random intercepts accounted for repeated pregnancies, and adjusted risk ratios (aRRs) were estimated controlling for key sociodemographic and clinical factors.

**Results:**

Structural poverty-related social determinants of health were associated with increased risk of adverse perinatal outcomes, including Black (aRR 1.50, 95% CI 1.42–1.59), Asian (aRR 1.49, 95% CI 1.39–1.59), and other minoritised ethnic backgrounds (aRR 1.50, 95% CI 1.42–1.59), residence in the most deprived areas (aRR 1.10, 95% CI 1.01–1.20), non-UK birth (aRR 1.20, 95% CI 1.15–1.25), and recent migration (aRR 1.32, 95% CI 1.14–1.53). Intermediary poverty-related social determinants of health were independently associated with increased risk beyond ethnicity and deprivation, including lack of social support (aRR 1.21, 95% CI 1.02–1.42), unemployment (aRR 1.16, 95% CI 1.10–1.23), financial hardship (aRR 1.17, 95% CI 1.01–1.35), living in social housing (aRR 1.16, 95% CI 1.09–1.24), transfer of care between hospitals (aRR 1.27, 95% CI 1.18–1.37), missed appointments (aRR 1.19, 95% CI 1.04–1.37), and unscheduled maternity care use (aRR 1.21, 95% CI 1.14–1.29). Women exposed to multiple overlapping poverty-related social determinants of health had a substantially higher likelihood of adverse perinatal outcomes (aRR 1.23, 95% CI 1.12–1.35).

**Conclusions:**

Structural and intermediary social determinants related to poverty have a substantial and cumulative impact on maternal and perinatal outcomes, independent of individual clinical risk. Addressing these inequities requires integrated, cross-sector strategies that extend beyond healthcare to the wider social conditions influencing maternal and child health.

**Clinical trial number:**

Not applicable.

**Supplementary information:**

The online version contains supplementary material available at 10.1186/s12939-026-02793-3.

## Background

Health inequities remain a persistent issue in the United Kingdom, disproportionately affecting marginalised populations, particularly in maternity care. The World Health Organization (WHO) defines health inequities as avoidable, unjust differences in health outcomes that arise from systemic social disadvantages [1]. In maternal and neonatal health, these inequities manifest as differences in maternal morbidity and mortality, preterm birth, low birthweight, stillbirth, and neonatal mortality, which can have lasting consequences for child development and intergenerational health.

In the UK context, these disparities are closely tied to social determinants of health (SDOH), including socioeconomic status, housing security, education, and systemic discrimination [2]. While the National Health Service (NHS) offers free maternity care to those deemed “ordinarily resident,” barriers such as complex eligibility rules, language difficulties, and limited culturally sensitive care continue to restrict equitable access, particularly for migrant and socioeconomically disadvantaged women [3].

Despite the NHS’s commitment to advancing health equity [4], substantial inequalities in maternal and infant outcomes persist across ethnic and socioeconomic groups. Research in the UK demonstrates that women from Black, Asian, and other minoritised ethnic backgrounds, as well as those born outside the UK or requiring interpreter support, experience higher risks of adverse maternal outcomes such as emergency caesarean and obstetric haemorrhage, as well as infant outcomes including preterm birth, low birthweight, low Apgar scores, and stillbirth or neonatal death, compared with White or UK-born women [5, 6]. Women living in more deprived areas also face elevated risks of these outcomes, illustrating the independent contribution of socioeconomic disadvantage [7–9]. Importantly, risks are greatest among women with intersecting vulnerabilities, highlighting persistent and complex inequalities in perinatal health that cannot be fully explained by single demographic factors alone [10–12]. These patterns underscore the need to examine broader social determinants of health to inform targeted, cross-sector interventions.

Although national policy increasingly acknowledges these inequities [13], there remains a paucity of UK-based quantitative research examining the full range of social determinants that shape maternity care access and outcomes. Particularly underexplored are structural and intermediary factors such as housing insecurity, economic precarity, immigration status, legal barriers to healthcare, and limited health literacy, which interact to shape both maternal and infant health risks [14–16]. Traditional efforts to reduce health disparities in high-income countries have often focused on improving healthcare access. However, research indicates that access alone accounts for only a modest share of health outcomes [17]. Structural determinants, including poverty, exert a more profound influence, through unstable living conditions, food insecurity, and cumulative psychosocial stress [8, 18, 19]. Yet, UK policy and research often rely on aggregated proxies, such as ethnicity or area-level deprivation, which obscure individual-level variation and limit the ability to examine the specific social determinants that contribute to maternal and neonatal inequities [12, 20–22].

**Fig. 1:**
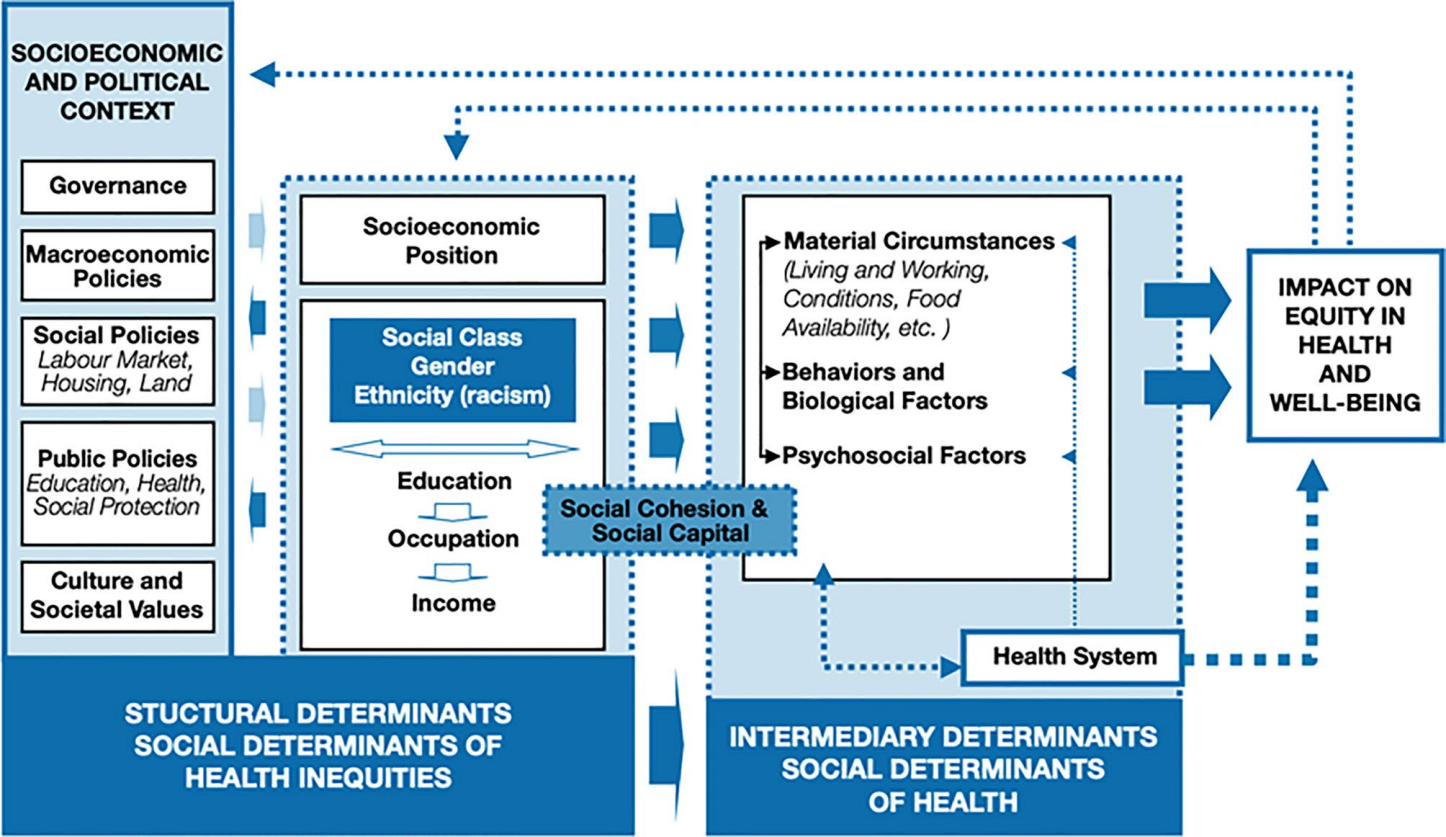
A conceptual framework for action on the social determinants of health [1]

The WHO Commission on Social Determinants of Health (CSDH) framework (see Fig. 1) provides a useful model for understanding how poverty and other social disadvantages influence health inequities. The framework distinguishes between structural determinants (e.g., low income, limited education, ethnicity), which shape the broader distribution of power, resources, and opportunity, and intermediary determinants (e.g., housing quality, employment insecurity, financial hardship, social support, health behaviours, healthcare access), which represent the more immediate conditions of daily life that directly influence health outcomes [1]. In this framework, poverty operates through both structural and intermediary determinants: for example, low income and employment insecurity can limit access to adequate housing, nutritious food, or healthcare, while systemic discrimination may exacerbate these disadvantages. Applying this framework in the UK maternity context enables a clearer understanding of how poverty and intersecting social disadvantages contribute to perinatal health inequities

This study investigates the association between poverty-related structural and intermediary social determinants and adverse perinatal outcomes in an ethnically diverse, urban area of the UK. Drawing on routinely collected local data, we apply the CSDH framework to identify key drivers of inequity and inform targeted, cross-sector strategies aimed at reducing maternal and child health disparities.

## Methods

### Aim

To examine the relationships between a composite adverse maternal outcome for mother and/or baby and poverty related social determinants of health within a South London cohort.

### Design

This study was a retrospective cross-sectional analysis of The Early Life Cross Linkage in Research (eLIXIR) cohort. The eLIXIR Partnership, established in 2018, generates a repository of real-time, pseudonymised, structured data derived from the electronic health record systems of two acute and one Mental Health NHS Trust in South London, UK [23]. Data are obtained under an opt-out consent model, meaning all individuals who do not opt out are included.

All women who gave birth to a singleton infant within the eLIXIR cohort were eligible for inclusion, with no maternal age restrictions. Information on congenital anomalies was not available, and maternal SARS-CoV-2 infection was not included as a covariate in this analysis, though it is examined in other eLIXIR work [24]. Sociodemographic characteristics and outcomes were derived from routinely collected electronic health records; further details on cohort construction and data linkage are available via the eLIXIR project [25].

The study included pregnancies from October 2018 to October 2023. Multiple (twins, triplets etc) pregnancies were excluded due to their intrinsically higher risk of obstetric and neonatal complications, which could obscure associations with poverty-related social determinants. Women were not excluded on the basis of late initiation of antenatal care; pregnancies with a first antenatal (booking) appointment at any gestational age were eligible for inclusion. Only completed singleton pregnancies with both a recorded booking appointment and a recorded birth at participating NHS Trusts were retained. Pregnancies without booking or birth data primarily reflect transfers of care or emergency presentations and could not be reliably analysed, as exposures were measured at booking and outcomes at birth.

### Setting

The study was conducted in an urban area of South London with high levels of socioeconomic deprivation and a large proportion of residents from minority ethnic and migrant backgrounds. Maternity care in this area is delivered through a network of NHS providers, including community, primary, secondary, and tertiary services. For the eLIXIR cohort, linked electronic maternity health records were obtained from two acute NHS Trusts and one mental health NHS Trust, covering pregnancies from booking appointment through to delivery. Postnatal follow-up, including health visitor data, was not included in this analysis. The dataset captures detailed maternal and infant clinical data, demographic characteristics, and social risk factors recorded during pregnancy and at delivery [23].

### Outcome measures

Adverse maternal and infant outcomes were defined based on the National Maternity and Perinatal Audit [26] and the English Maternal Morbidity Outcome Indicator [27]. Outcome definitions were translated to the eLIXIR dataset using available data fields and validated clinical codes (ICD-10 for diagnoses, OPCS-4 for procedures). The primary outcome was a composite binary variable indicating the presence of any of the following: emergency (unplanned) caesarean section, obstetric haemorrhage exceeding 499 mL (antepartum or postpartum), preterm birth (<37 weeks gestation), low birthweight (<2500 grams), low Apgar score (≤7 at 5 minutes), stillbirth (intrauterine death ≥24 weeks gestation), or neonatal death within 28 days of birth. All outcomes were coded as binary variables. Full definitions, data sources, and clinical codes are provided in Supplementary File 1.

#### Poverty related social determinant variables

We applied the World Health Organization Commission on Social Determinants of Health (CSDH) framework to guide the selection and classification of poverty-related variables available in the eLIXIR dataset. The framework distinguishes between:

- **Structural determinants**, reflecting individuals’ social position and systemic disadvantage (e.g. education, income, ethnicity, legal status); and
- **Intermediary determinants**, capturing material circumstances, psychosocial stressors, behavioural factors, and interactions with the health system (World Health Organization (WHO), 2010).

Variables were included if they represented dimensions of socioeconomic disadvantage plausibly influencing maternal or perinatal outcomes. All relevant variables in the eLIXIR dataset were mapped to the CSDH framework (Table 1; full definitions in Supplementary File 1). This mapping ensured that each variable could be explicitly linked to poverty-related disadvantage and supported interpretation of potential targets for intervention.

**Table 1:** Poverty-related social determinant variables available in eLIXIR, mapped to the WHO CSDH framework

CSDH Domain	Variables Available in eLIXIR
Structural Determinants	Ethnicity (non-white, as a proxy for structural racism and marginalisation); Deprivation index (two highest quintiles, reflecting socioeconomic disadvantage); Age < 20 (young maternal age, associated with socioeconomic vulnerability); Born outside UK; Refugee/asylum seeker; New to country; No right to work; Financial difficulties; Social care involvement (reflecting systemic disadvantage); Unborn child subject to foster care/adoption; Criminal justice involvement; Female genital mutilation (marker of cultural marginalisation and structural inequities)
Intermediary Determinants	Interpreter required (reflecting language barriers and access to care); Feels unsupported (low social support, psychosocial stressor); Housing issues or homelessness; Substance use; Domestic abuse (past or current); Mental health problems; Learning difficulties (associated with educational disadvantage and socioeconomic vulnerability); Late booking for maternity care; Transfer of care from another hospital; Missed > 2 antenatal appointments; Inadequate antenatal care; Unscheduled maternity care use; A&E visits during pregnancy
Both Structural and Intermediary	Multiple risk factors composite variable (indicating co-occurrence of three or more poverty-related social determinants, capturing cumulative disadvantage)

Some variables span both structural and intermediary domains. For example, homelessness, criminal justice involvement, and social care engagement reflect broader structural disadvantage and economic precarity, while also directly shaping daily stress, housing instability, and access to healthcare. Learning difficulties and language barriers were not conceptualised as being caused by poverty. Rather, they were classified as intermediary determinants that frequently co-occur with socioeconomic disadvantage and constrained access to resources. Within the CSDH framework, these factors influence individuals’ capacity to access, navigate, and benefit from healthcare, thereby mediating the relationship between underlying structural disadvantage and health outcomes. They were therefore treated as poverty-related social determinants insofar as they contribute to the persistence of socioeconomic inequalities in access to care and outcomes.

Intersectional disadvantage was defined using a binary indicator denoting exposure to three or more poverty-related social determinants, capturing the cumulative impact of multiple, intersecting dimensions of socioeconomic disadvantage.

### Analysis

Covariates were selected a priori based on existing literature and interdisciplinary clinical expertise, reflecting factors known to influence maternal and perinatal outcomes. Patterns of missing data were examined and confirmed to be missing completely at random using Little’s test [28], therefore, a complete case analysis was undertaken rather than multiple imputation. All analyses were conducted using R (version 4.3.1) to support transparency and reproducibility. Pregnancies with duplicate records or involving multiple gestations (e.g. twins) were excluded in line with the study eligibility criteria.

Poisson regression models with a random intercept for individual woman ID were used to account for multiple pregnancies per woman. Models estimated associations between each poverty-related social determinant and the composite adverse maternal outcome, adjusting for relevant maternal sociodemographic and clinical characteristics, including socioeconomic deprivation (Index of Multiple Deprivation), maternal age (as a confounder), parity, smoking status, body mass index > 30 kg/m^2^, pre-existing medical conditions, previous caesarean section, and previous preterm birth (all established risk factors for adverse outcomes [29–31]. Pre-existing medical conditions reflect clinician-assessed obstetric or medical risk identified at the initial maternity booking appointment and recorded as a binary risk designation within the electronic health record, rather than individual diagnostic codes (see Supplementary Table 1 for variable definitions). Models did not adjust for maternal ethnicity, as ethnicity is strongly correlated with structural disadvantage; adjusting for it could attenuate or obscure associations between poverty-related social determinants and outcomes. Risk ratios (RRs) with 95% confidence intervals (CIs) were reported, and statistical significance was defined as *p* ≤ 0.05.

Formal mediation analysis was not conducted because temporal ordering of exposures and outcomes could not be fully established, and some intermediary variables were incompletely reported. Instead, Specification Curve Analysis (SCA) was employed to address the wide range of plausible analytic decisions and the complex interrelationships among psychosocial, behavioural, and demographic predictors of harm. Traditional regression approaches typically rely on a single model specification, which may obscure sensitivity to researcher degrees of freedom [32]. SCA mitigates selective reporting by systematically estimating all plausible model specifications across combinations of exposures, covariates, and outcomes, and summarising the resulting distribution of effect estimates.

## Results

**Fig. 2:**
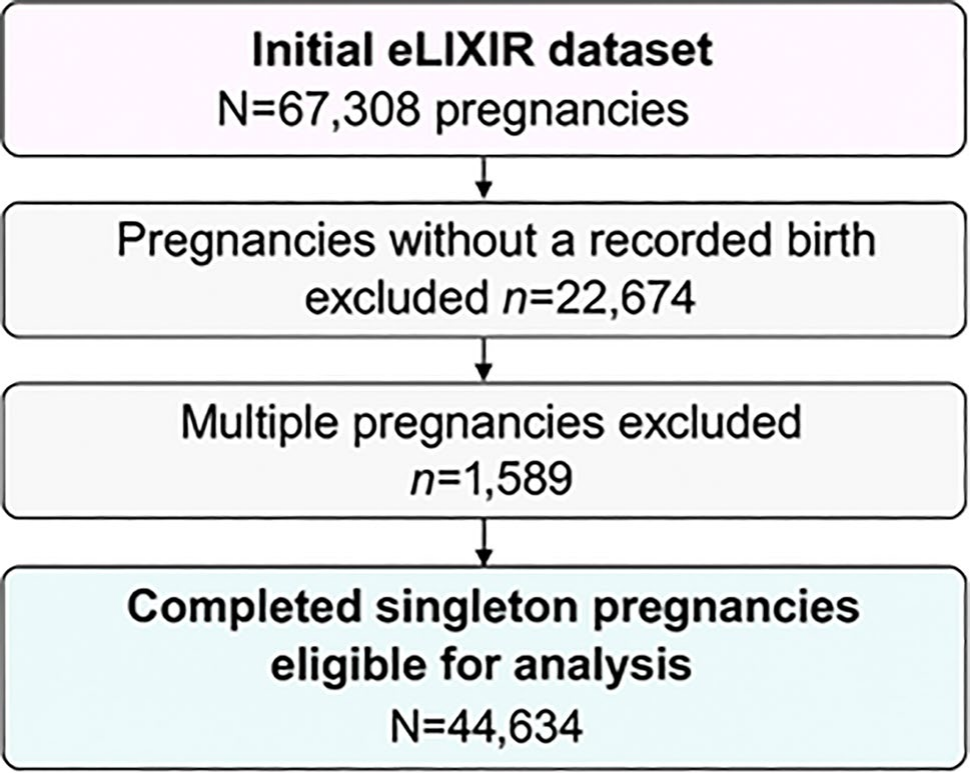
Cohort flow diagram showing the removal of participants to the final sample for analysis

The initial eLIXIR dataset included 67,308 pregnancies from 56,426 women. After applying eligibility criteria, pregnancies without a recorded birth (*n* = 22,674) and multiple pregnancies (*n* = 1589) were excluded, leaving 44,634 completed singleton pregnancies for analysis. A total of 15,633 (35.2%) adverse maternal or infant outcomes were recorded across the retained pregnancies (see Fig. 2 for cohort flow diagram).

### Participant characteristics

Table 2 outlines the maternal baseline characteristics at the time of the booking appointment. The majority of the sample identified their ethnicity as White (53.41%), followed by Black (19.92%), Asian British (10.08%), Mixed/Multiple ethnic groups (5.21%), and other ethnic groups (6.53%), with 4.85% of ethnicity data missing. Nearly three quarters (74.42%) of participants were classified as having a high medical risk status at booking, and 46.21% were primiparous. The mean maternal age at booking was 32.86 years (SD = 5.42), the average BMI was 24.39 (SD = 6.63), and 3.77% of women reported smoking at the time of booking.

**Table 2:** Maternal baseline characteristics and disaggregated outcomes

Demographic	n(%)
Age at booking (years) mean ±SD	32.86 (5.42)
Primiparous	23313 (52.23%)
High medical risk status at booking	33219 (74.42%)
BMI	24.39 (SD 6.63)
Smoker at booking	1684 (3.77%)
**Birth outcome**	
Emergency (unplanned) caesarean section,	10688 (24.09%)
Obstetric haemorrhage > 1000 ml	4970 (11.2%)
Preterm birth < 37/40	4009 (9.04%)
Low birthweight < 2500 grams	4152 (9.36%)
Low Apgar score (<7 at 5 minutes)	777 (1.75%)
Stillbirth or Neonatal Death	502 (1.13%)

### Structural determinants

Table 3 presents the risk ratios (RR) and adjusted risk ratios (aRR) for the composite measure of adverse outcomes, stratified by structural, intermediary, and intersectional determinants of health, and figure two presents a forest plot of any adverse outcome by social determinant.

**Table 3:** Social determinant/risk factor RRs and aRrs for any adverse outcome (composite measures combined)

	Total number 44634 n(%)	Adverse outcome 15633 n(%)	aRR*95% CI
Ethnicity			
White Any other Black Mixed/Multiple Asian Missing	23838 (53.41%) 2916 (6.53%) 8890 (19.92%) 2326 (5.21%) 4501 (10.08%) 2163 (4.85%)	7686 (49.17%) 1041 (6.66%) 3516 (22.49%) 807 (5.16%) 1831 (11.71%) 752 (4.81%)	ref (1) 1.25 (1.14, 1.36)*** 1.61 (1.52, 1.70)*** 1.21 (1.10, 1.33)*** 1.53 (1.43, 1.64)***
Deprivation quintile			
1^st^ (most deprived) 2^nd^ 3^rd^ 4^th^ 5^th^ (least deprived) Missing	8155 (18.27%) 16728 (37.48%) 10474 (23.47%) 5321 (11.92%) 3176 (7.12%) 780 (1.75%)	2950 (18.87%) 5853 (37.44%) 3553 (22.73%) 1834 (11.73%) 1088 (6.96%) 355 (2.27%)	1.17 (1.07, 1.28)* 1.09 (1.01, 1.19) 1.00 (0.92, 1.09) 1.01 (0.92, 1.09) ref (1) -
Age < 20 at time of birth			
No Yes	44091 (98.78%) 543 (1.22%)	15455 (98.86%) 178 (1.14%)	ref (1) 0.91 (0.5479)***
Born outside UK			
No Yes	20470 (45.86%) 24164 (54.14%)	6809 (43.56%) 8824 (56.44%)	ref (1) 1.20 (1.15, 1.25)***
Refugee or Asylum Seeker			
No Yes	44215 (99.06%) 419 (0.94%)	15480 (99.02%) 153 (0.98%)	ref (1) 1.22 (0.99, 1.50)
New to country			
No Yes	43841 (98.22%) 793 (1.78%)	15291 (97.81%) 342 (2.19%)	ref (1) 1.43 (1.24–1.67)***
No right to work			
No Yes Missing	43434 (97.31%) 182 (0.41%) 1018 (2.28%)	15152 (96.92%) 56 (0.36%) 425 (2.72%)	ref (1) 0.85 (0.61, 1.16) -
Financial difficulties			
No Yes Missing	32006 (71.71%) 866 (1.94%) 11762 (26.35%)	11010 (70.43%) 331 (2.12%) 4292 (27.45%)	ref (1) 1.23 (1.07, 1.44)** -
Social Care involvement (current or previous)			
No Yes	43532 (97.53%) 1102 (2.47%)	15235 (97.45%) 398 (2.55%)	ref (1) 1.27 (1.11, 1.46)***
Criminal justice involvement			
No Yes Missing	−42903 (96.12%) 884 (1.98%) 847 (1.90%)	14944 (95.59%) 313 (2.00%) 376 (2.41%)	ref (1) 1.07 (0.92, 1.23) -
Interpreter required			
No Yes Missing	39482 (88.46%) 3078 (6.90%) 2074 (4.65%)	13785 (88.18%) 1074 (6.87%) 774 (4.95%)	ref (1) 1.13 (1.04, 1.23)** -
Feels unsupported			
No Yes	43992 (98.56%) 642 (1.44%)	15384 (98.41%) 249 (1.59%)	ref (1) 1.21 (1.02, 1.42)*
Homeless/Housing Issues			
No Yes	37936 (84.99%) 1991 (4.46%)	13232 (84.64%) 715 (4.57%)	ref (1) 1.10 (0.99, 1.21)
Social Housing			
No Yes Missing	36447 (81.66%) 6106 (13.68%) 2081 (4.66%)	12681 (81.12%) 2107 (13.48%) 845 (5.41%)	ref (1) 1.16 (1.09, 1.24)*** -
Unemployment (exc no right to work)			
No Yes Missing	37372 (83.73%) 6244 (13.99%) 1018 (2.28%)	12915 (82.61%) 2293 (14.67%) 425 (2.72%)	ref (1) 1.26 (1.19, 1.35)***
Substance use			
No Yes Missing	40802 (91.41%) 3826 (8.57%) 6 (0.01%)	14158 (90.56%) 1472 (9.42%) 3 (0.02%)	ref (1) 1.05 (0.97, 1.13) -
Domestic abuse (previous and current)			
No Yes Missing	34992 (78.40%) 251 (0.56%) 35 (0.08%)	12131 (77.60%) 109 (0.70%) 7 (0.04%)	ref (1) 1.61 (1.22, 2.11)*** -
Current mental health issues			
No Yes	33431 (74.90%) 11203 (25.10%)	11675 (74.68%) 3958 (25.32%)	Ref (1) 0.99 (0.95, 1.04)
Referred to mental health services during pregnancy			
No Yes Missing	42824 (95.94%) 1751 (3.92%) 59 (0.13%)	14946 (95.61%) 657 (4.20%) 30 (0.19%)	ref (1) 1.14 (1.02, 1.26)* -
Previous mental health inpatient admission			
No Yes Missing	37560 (84.15%) 7015 (15.72%) 59 (0.13%)	13168 (84.23%) 2435 (15.58%) 30 (0.19%)	Ref (1) 1.01(96, 1.07) -
Learning difficulties			
No Yes	43784 (98.10%) 850 (1.90%)	15296 (97.84%) 337 (2.16%)	Ref (1) 1.21 (1.04, 1.40)*
Late maternity care booking > 13/40			
No Yes	37225 (83.40%) 7409 (16.60%)	13062 (83.55%) 2571 (16.45%)	Ref (1) 0.97 (0.92, 1.02)
Late maternity care booking > 20/40			
No Yes	44334 (99.33%) 300 (0.67%)	15519 (99.27%) 114 (0.73%)	Ref (1) 1.10 (0.85, 1.41)
Transfer of care from another hospital			
No Yes	41140 (92.17%) 3494 (7.83%)	14150 (90.51%) 1483 (9.49%)	Ref (1) 1.22 (1.13, 1.32)***
Missed antenatal appointments > 3			
No Yes	43647 (97.79%) 987 (2.21%)	15273 (97.70%) 360 (2.30%)	Ref (1) 1.31 (1.13, 1.50)***
Inadequate AN care (<10 for primips, <7 for multips)			
No Yes	39843 (89.27%) 4791 (10.73%)	13830 (88.47%) 1803 (11.53%)	Ref (1) 1.01 (0.95, 1.09)
Unscheduled access to maternity care (maternity triage)			
No Yes	6491 (14.54%) 38143 (85.46%)	1934 (12.37%) 13699 (87.63%)	Ref (1) 1.25 (1.17, 1.33)***
Multiple risk factors > 3 of any of the above			
No Yes	42632 (95.51%) 2002 (4.49%)	14808 (94.72%) 825 (5.28%)	Ref (1) 1.27 (1.15, 1.39)***

Absence of ‘missing’ numbers indicate no missing data. Adjusted for parity, pre-existing medical risk, previous pre-term birth, previous caesarean section, maternal age, BMI>/30 kg/m2 and smoker at booking

***<0.001, **<0.01, *<0.05

Women from racially minoritised groups experienced consistently higher risks of adverse outcomes compared to White women. Black and Asian women had approximately 50% increased adjusted risk (Black: aRR = 1.61, 95% CI: 1.52–1.70; Asian: aRR = 1.53, 95% CI: 1.43–1.64), while Mixed ethnicity and Other ethnic backgrounds also showed elevated but smaller increase risk (aRRs between 1.21 and 1.25). Women in the most deprived quintile experienced an 17% higher adjusted risk of adverse outcomes compared to those in the least deprived areas (aRR = 1.17, 95% CI: 1.07–1.28), suggesting socioeconomic context as a determinant of perinatal risk.

Younger women (<20 years) were associated with improved outcomes, analysis indicated they were 35% less likely to experience adverse events (aRR = 0.65, 95% CI: 0.54–0.79).

Being born outside the UK conferred a 20% higher adjusted risk (aRR = 1.20, 95% CI: 1.15–1.25), and those newly arrived in the UK had a 43% increased risk (aRR = 1.43, 95% CI: 1.24–1.67) of an adverse outcome. Financial difficulties were associated with a 17% elevated risk (aRR = 1.17, 95% CI: 1.01–1.35).

Missing more than three antenatal appointments was associated with a 31% increased adjusted risk (aRR = 1.31, 95% CI: 1.13–1.50). Similarly, unscheduled access to maternity care (e.g., triage visits) was linked with a 25% higher risk (aRR = 1.25, 95% CI: 1.17–1.33). In contrast, inadequate antenatal care by appointment count showed no association after adjustment (aRR = 1.01, 95% CI: 0.95–1.09), suggesting that frequency alone may not capture care quality or continuity.

### Intermediary determinants

Psychosocial and economic stressors were linked to elevated risk. Women reporting a lack of support had a 21% increased adjusted risk of adverse outcomes (aRR = 1.21, 95% CI: 1.02–1.42), and unemployment was similarly associated with increased risk (aRR = 1.26, 95% CI: 1.19–1.35). Domestic abuse showed one of the strongest associations among intermediary factors, with a 61% higher adjusted risk (aRR = 1.61, 95% CI: 1.22–2.11).

Living in social housing was associated with a 16% higher risk (aRR = 1.16, 95% CI: 1.09–1.24), reflecting broader structural disadvantage. Other indicators such as substance use, current mental health issues, or referral to mental health services during pregnancy did not show significant associations.

Learning difficulties and experiences of homelessness or housing insecurity showed modest or uncertain associations, with confidence intervals crossing the null. Similarly, interpreter use and late booking for maternity care (>13 or > 20 weeks) were not independently associated with adverse outcomes in adjusted analyses. However, transfer of care between hospitals was associated with a 22% increased risk (aRR = 1.22, 95% CI: 1.13–1.32), indicating the potential impact of discontinuity in maternity care.

### Intersectional determinants

Women with more than three co-occurring social or health-related risk factors (excluding ethnicity and deprivation) had a 27% increased adjusted risk of experiencing an adverse outcome (aRR = 1.27, 95% CI: 1.15–1.39).

### Specification curve analysis

**Fig. 3:**
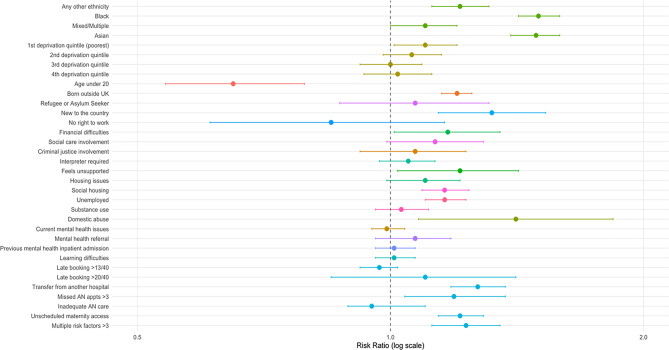
Forest plot of any adverse outcome by social determinant

**Fig. 4:**
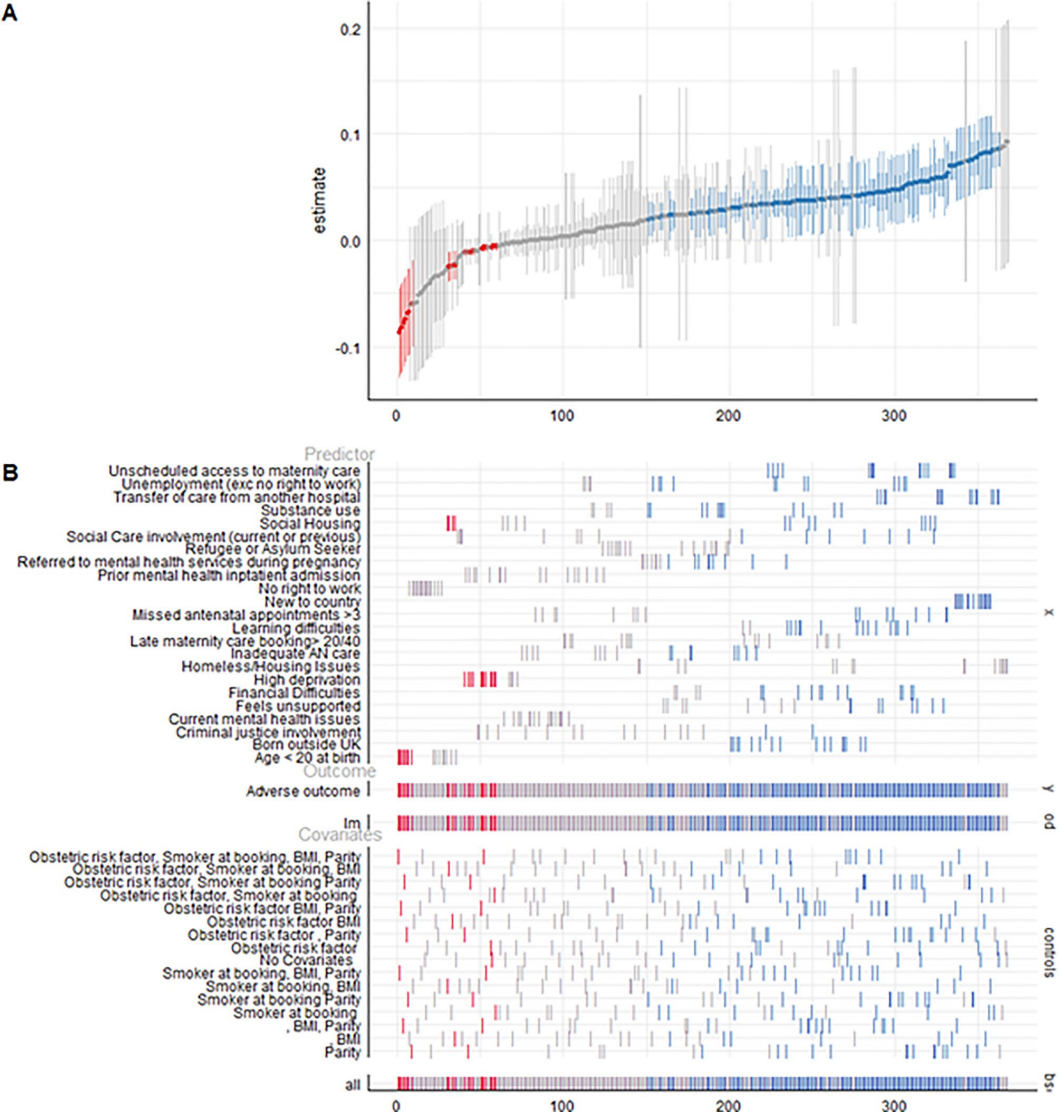
Panel a shows effect estimates and 95% confidence intervals across 360 model specifications. Significant negative associations (red) indicate reduced risk, and positive associations (blue) indicate increased risk of adverse outcomes, assuming higher outcome values reflect greater adversity. Panel B maps the included predictors, outcome, and covariates for each model, illustrating the variation across the specification space

In the full sample (*n* = 432 specifications), the median effect estimate for the association between social risk factors and adverse outcomes 0.03, with an interquartile range (IQR) of 0.00 to 0.04. The median R^2^ across models was 0.013, indicating that predictors accounted for 1.3% of variance in adverse outcomes. Several predictors showed significant positive associations with the adverse outcome, including substance use, social housing, social care involvement, refugee or asylum seeker status, prior mental health admission, no right to work, and housing instability, indicating a increased risk of adverse outcomes when these factors are present (see Fig. 3). Conversely, women under 20 at birth, and criminal justice involvement were significantly negatively associated with adverse outcomes, suggesting an reduced risk in these groups. (Fig. 4).

## Discussion

This study highlights the significant influence of socioeconomic and social determinants on maternal and perinatal outcomes in a large, ethnically diverse urban UK cohort. By mapping routinely recorded social risk factors onto the WHO Commission on Social Determinants of Health (CSDH) framework [1, 7], we demonstrate how structural determinants, such as deprivation, minoritisation, and immigration status, and intermediary determinants, including housing instability, social isolation, mental health, and service access, independently and cumulatively contribute to elevated risk. Associations persisted after adjusting for clinical risk factors, reinforcing the intersectional nature of maternal health vulnerabilities, where multiple disadvantages interact to exacerbate outcomes beyond the sum of individual factors (10–12].

Certain groups experienced particularly elevated risk. Women from Black, Asian, and minority ethnic backgrounds continued to face disproportionately poor outcomes, even after accounting for area-level deprivation, highlighting how ethnicity intersects with socioeconomic and systemic disadvantage [7, 9, 12, 33]. Migrant women also faced higher risk, likely reflecting barriers related to immigration status, limited entitlement to care, language proficiency, and unfamiliarity with healthcare systems, all of which may be addressed through more inclusive policy and service design [14, 34]. Consistent with this, findings from another eLIXIR-BiSL study [33] identified a disproportionate risk of adverse maternal and infant outcomes where minority ethnicity and foreign-born status co-occurred.

Exposure to domestic abuse during pregnancy was strongly associated with adverse outcomes, interacting with other social determinants such as socioeconomic disadvantage and lack of support, illustrating layered vulnerability [35]. While NHS guidelines exist for identifying and managing domestic abuse [36], our study did not assess their implementation or effectiveness. These findings highlight the importance of early identification, trauma-informed care, and integrated support services to mitigate layered risks.

Interestingly, younger maternal age (<20 years) was associated with lower odds of adverse outcomes. While younger age is often considered a risk factor [37], this finding may reflect several protective factors in this population. Teenagers are less likely to have chronic conditions or complex medical histories, and targeted support services for teenage or first-time mothers may provide additional benefit [38]. These lower odds are likely driven by lower rates of emergency caesarean section, which was a key component of our composite adverse outcome. Evidence suggests that teenagers experience higher rates of unassisted vaginal births, potentially due to intrinsic biological factors such as greater uterine contractility, with caesarean indications like abnormal fetal presentation representing a minority [39]. Family and social support may further buffer risk. These results illustrate that younger maternal age does not uniformly predict adverse outcomes and highlight the need for further research into the interplay of biological, social, and service-related factors to avoid assumptions that could lead to misdirected resource allocation.

Many sensitive social risks, including domestic violence, substance use, and mental health challenges, are often not disclosed at initial booking appointments due to stigma, fear of repercussions, or concerns about social care involvement. When maternity services are perceived as surveillance rather than support, disclosure may be further inhibited. Continuity of care can mitigate these barriers by fostering trust-based relationships and enabling holistic, individualised care that responds to women’s needs over time [40–42]. Structured tools such as MATDAT [43] and holistic cross sectoral approaches to identifying social risk [44] have been developed to support more systematic identification, though full evaluation is required before scale up. Expanding data collection to capture underrepresented domains such as housing precarity, language barriers, and cumulative trauma, alongside intersectional and mixed methods research, remains important for strengthening understanding and care.

Beyond risk identification, recognising and integrating community strengths and resilience is essential. Social capital, informal support networks, faith groups, and culturally rooted knowledge systems can sustain maternal mental health and parenting under adversity. Embedding these assets within community based and continuity of care models can enhance equity and cultural responsiveness, with emerging evidence of benefit for maternal and infant health inequalities [5, 10, 16, 30, 45–47, 48, 49].

For migrant women, Stevenson et al. [30] highlight that perinatal care can be strengthened through multidisciplinary teams with access to specialist in person interpreters, maternal education programmes, and integrated social support including housing, immigration, food, and essential baby items. Group antenatal care, mental health support, and continuity with known midwives or small teams appear particularly effective. Evidence synthesised by Vousden et al. [16] further suggests that home based, psychosocial, maternity care models and interdisciplinary programmes may benefit maternal and child health when they address advocacy, support, and information needs while accounting for culture, past trauma, and upstream determinants such as housing and finances. Together, these findings underscore the importance of aligning policy, public health, and clinical approaches to address maternal and infant inequalities within high income health service contexts.

## Strengths and limitations

This study draws on a large, diverse urban cohort with linked clinical, social, and demographic data, enabling robust examination of multiple intersecting social determinants of perinatal outcomes. All women attending antenatal booking appointments, including those booking later in pregnancy, and those with recorded birth outcomes were included, reducing the risk of selection bias and supporting the generalisability of observed associations. The use of routinely collected data enhances relevance to policy and practice, and Specification Curve Analysis strengthens confidence in the robustness of findings across multiple analytic decisions [32, 50].

Several limitations should be acknowledged. Routine health data do not capture nuanced determinants such as discrimination, social class, acculturation, or trauma, introducing residual confounding and likely underestimating social complexity. Sociodemographic variables such as ethnicity and area-level deprivation are aggregated, and reporting of sensitive information (e.g., language proficiency, immigration status, mental health, domestic violence) may be incomplete or inconsistent, potentially affecting reliability at the individual level. Conceptual overlap between structural and intermediary determinants may lead to over-adjustment; although Specification Curve Analysis helps address this, adjusted estimates should still be interpreted cautiously. Pregnancies with missing key exposure or outcome data were excluded, and complete case analysis was used rather than multiple imputation. Little’s test [24] indicated that missingness was consistent with missing completely at random, and comparison with available data suggested excluded cases were broadly similar to included cases, supporting robustness. Models were adjusted for key prior adverse outcomes where available; adjustment for prior stillbirth was not possible. Findings represent associations within a complex system of interrelated social and clinical determinants rather than causal effects.

The composite adverse outcome includes emergency caesarean section and preterm birth, which may represent clinically appropriate interventions rather than adverse events in all cases; future research could disaggregate these outcomes to better elucidate underlying mechanisms. As an observational study, causal inference is limited. Findings should be interpreted as associations within a complex system of interrelated social and clinical determinants.

## Conclusion

This study highlights the complex and multifaceted impact of social determinants on maternal and perinatal outcomes in a diverse UK urban population. Both structural factors, such as deprivation, immigration status, and ethnicity, and intermediary determinants, including housing instability, social support, and access to services, interact to shape health risks. Less easily measured influences, such as social class, acculturation, and prior traumatic experiences, are also likely to contribute and warrant further investigation.

Recognising and integrating community strengths and resilience, including social networks and cultural resources, into maternity care is essential for equitable and effective intervention. Promising findings from community-based continuity of care models suggest that sustained, relationship-based care can improve engagement, support disclosure of social risk, and contribute to improved maternal and perinatal outcomes, particularly for women experiencing multiple forms of disadvantage. Our findings support the need for early, multisectoral collaboration, the development and evaluation of tools for the systematic identification of social risks, and policies that aim to reduce poverty while providing financial and relational support to those at increased risk. Adopting holistic life course approaches and learning from international best practice may help shift maternity care beyond a narrow biomedical focus to more comprehensively address social determinants, with the ultimate goal of reducing perinatal health inequalities.

## Electronic supplementary material

Below is the link to the electronic supplementary material.

Supplementary Material 1

## Data Availability

The data accessed by eLIXIR remain within an NHS firewall and governance is provided by the eLIXIR Oversight Committee reporting to relevant information governance clinical leads. Subject to these conditions, data access is encouraged and those interested should contact the eLIXIR Chief Investigator (Professor Lucilla Poston; Lucilla.poston@kcl.ac.uk).
