# Supplementary material for: The role of poverty-related social determinants in maternal and perinatal health inequities: a cross-sectional study using the eLIXIR born in South London, UK maternity-child data linkage
Source: Int J Equity Health. 2026 Mar 14;25:109. doi: 10.1186/s12939-026-02793-3 (PMC13101210; doi:10.1186/s12939-026-02793-3)
Supplement: Supplementary file 1 — Supplementary Material 1 [file 12939_2026_2793_MOESM1_ESM.docx]

| **Supplementary Table S1: Outcome measure, corresponding database variables and definitions** | | |
| --- | --- | --- |
| **Exposure/Outcome** | **Data set** | **Definition** |
| Adverse Outcome |  | Composite of emergency (or unplanned) caesarean section and/or obstetric (antepartum and/or postpartum) haemorrhage >1000mls, and/or preterm birth (<37 weeks), and/or low birthweight (<2500g), and/or Apgar score less than or equal to 7 at 5 minutes after birth, and/or neonatal death (within 28 days of birth), and/or still birth (death occurring before or during birth once a pregnancy has reached 24 weeks). |
| Emergency (or unplanned) caesarean section | DS2 Intrapartum and postnatal care | ModeOfDelivery = “Emergency an unspecified caesarean section” |
| Obstetric haemorrhage | DS2 Intrapartum and postnatal care | TotalBloodLoss >499ml |
| Pre-term birth | DS2 Intrapartum and postnatal care | GestationAtDeliveryWeeks <37 |
| Low Birth Weight | DS2 Intrapartum and postnatal care | BirthWeightGrams<2500g |
| Low Apgar Score | DS2 Intrapartum and postnatal care | APGARscore5minutes<7 at 5 minutes after birth |
| Neonatal death of stillbirth | DS2 Intrapartum and postnatal care | FinalBirthOutcome = “Livebirth” |
| Ethnicity  White  Any other  Black  Mixed/multiple  Asian | DS1 Booking dataset | MotherEthnicity categorised based off of ONS broad categories  Individuals who did not fall into the categories based off of ONS broad definitions, were categorised as “Any other” if they were categorised in the NHS data set as “Any Other ethnic group” |
| High Medical risk at booking | DS1 Booking dataset | Free text search of MedicalRiskFactor for any medical risk factors identified at booking appointment (e.g. neurological condition). |
| BMI | DS1 Booking dataset | BMIAtBooking |
| Smoker at booking | DS1 Booking dataset | SmokerAtBooking,=”Yes” or “No” |
| **Deprivation quintile**  1^st^ (most deprived)  2^nd^  3^rd^  4^th^  5^th^ (least deprived)  Missing | DS1 Booking dataset | IMDQuintile |
| **Age < 20 at time of birth** | DS1 Booking dataset | MaternalAgeAtBooking<20 years |
| Born outside UK | DS1 Booking dataset | CountryofBirth not “United Kingdom or Great Britain” |
| **Refugee or Asylum Seeker** | DS1 Booking dataset | Citizenship= “Refugee” or “Asylum Seeker” |
| **New to country** | DS1 Booking dataset | SocialRiskFactors= “New to country” |
| **No right to work** | DS1 Booking dataset | EmploymentStatus= “No rights to work” |
| **Financial difficulties** | DS8 Other Social Issues dataset | FinancialDifficulties=”Yes” or “No” |
| **Social Care involvement (current or previous)** | DS8 Other Social Issues dataset | PreviousSocialServicesInvolvement=”Yes” or “No”  &  HasSocialServicesInvolvement=”Yes” or “No” |
| **Criminal justice involvement (current or previous)** | DS8 Other Social Issues dataset | EverInvolvedCriminalJusticeSystem=”Yes” or “No |
| **Late maternity care booking >13/40** | DS1 Booking dataset | GestationAtBookingWeeks>13 weeks |
| **Late maternity care booking >20/40** | DS1 Booking dataset | GestationAtBookingWeeks>20 weeks |
| **Missed antenatal appointments >3** | DS4 Antenatal Appointment dataset | Attended=”DNA”  &  TypeOfAppointment was not “Induction” or “Labour” |
| **Inadequate AN care (<10 for primips, <7 for multips)** | DS4 Antenatal Appointment dataset | Attended=”Yes”<10 or <7  &  TypeOfAppointment was not “Induction” or “Labour” |
| **Unscheduled access to maternity care (maternity triage)** | DS3 MAU (Maternity Assessment Unit) dataset | ReasonForVisit |
| Interpreter required | DS1 Booking dataset | InterpreterRequired=”Yes” |
| **Feels unsupported** | DS1 Booking dataset | FeelSupported=”Yes” or “No” |
| **Homeless/Housing Issues** | DS8 Other Social Issues dataset | HousingPorblems=”Yes” or “No” |
| **Social Housing** |  |  |
| **Unemployment (exc no right to work)** | DS1 Booking dataset | EmploymentStatus= “Unemployed” |
| **Substance use** | DS1 Booking dataset | EverUsedDrugsOrSubstances=”Yes” or “No” |
| **Domestic abuse (previous and current)** | DS7 Domestic Violence dataset | PreviousDomesticAbuse=”Yes” or “No”  &  CurrentAbuseReferralRequired=”Yes” or “No” |
| **Current mental health issues** | DS1 Booking dataset | MentalHealthProblems |
| **Referred to mental health services during pregnancy** | Clinical Records Integration System, South London & Maudsley | Pregnancy_eJPS=”Yes” or ”No” |
| **Previous mental health inpatient admission** | Clinical Records Integration System, South London & Maudsley | Prior_inpatient=”Yes” or “No” |
| **Learning difficulties** | DS1 Booking dataset | SocialRiskFactors= “Learning Disabilities” |
